# Supplementary material for: A realist review of advance care planning for people with multiple sclerosis and their families
Source: PLoS One. 2020 Oct 16;15(10):e0240815. doi: 10.1371/journal.pone.0240815 (PMC7567361; doi:10.1371/journal.pone.0240815)
Supplement: S1 File — (DOCX) [file pone.0240815.s001.docx]

**S1 FILE: STUDY/LITERATURE CHARACTERISTICS**

| **Study** | **Aim** | **Article type** | **Main findings** | **Contribution to CMO** |
| --- | --- | --- | --- | --- |
| **Source articles focused on multiple sclerosis exclusively** | | | | |
| Buecken et al. (2012) [1] | To quantify level of patient desire to communicate about disease progression among PwMS and to identify role of physician empathy in discussing these concerns. | Quantitative study  N=573 | Most participants considered it important their doctor address progression of their disease and reach decisions with them concerning EoL situations. | **RELATIONSHIP**: Physician empathy plays a key role: “people suffering from longstanding illnesses regard physician empathy as particularly important” (p. 322)  **COMMUNICATION:** *“Communication about disease progression, including the discussion of ACP, is relevant to a vast majority of MS patients surveyed. The same data also point to a lack of actual communication”* (p. 322). Physicians and patients require training in EoL communication. |
| Chen & Habermann, (2013) [2] | To explore how couples living with advanced MS approach planning for health changes together. | Qualitative study (descriptive)  N=10 couples | Several activities helped couples prepare for the future: writing advance directives or living wills, communicating with loved ones about their future plans, planning to move to a more supportive living environment and receiving home health services ( p. 41). The partner with MS was the main health care decision-maker, but the spouse was vital during the decision-making process (p. 41). It was “a family affair” (p. 41). 3 types of dyads: i) making decisions up front; ii) on the to-do list; iii) hoping for a cure. (p. 41). | **PREVIOUS EXPERIENCE:** i) up front: 5 couples who had lived through close calls of the spouse had talked about what they might do if the MS spouse’s health worsened (p. 42).  ii) on the to do list: Four couples had done some planning but had not written ADs. The spouse had heard their partner with MS talk about worsening health, but they had not discussed healthcare preferences.  iii) hoping for a cure had no AD or living will written, had not communicated their wishes to loved ones and had not planned for a decline in health, including supportive living arrangements. Three of the 10 couples were in this category. In private, they had reflected on what might be coming as the MS progressed, but avoided entertaining those thoughts and had difficulty discussing feelings about the future, hoping for a cure (p. 43) Couples in this category all experienced some level of difficulty communicating. They were aware of the progressive nature of MS and thought about what the future may bring…. but preferred to focus on today (p. 43)  **AUTONOMY:** The one thread that ran through all these interviews was that of choice (p. 43). Care giving spouses wanted to support the ability of the spouse with MS to choose. Likewise, the spouse with MS didn’t want to feel that others were arbitrarily making choices for them (p. 43). |
| Col, et al. (2018) [3] | To evaluate the preference assessment tool in a national sample of PwMS. | Quantitative study  N=135  Part of a larger study to develop and evaluate a tool to assess patient goals and preferences for MS treatments | The preference assessment tool successfully captured patients’ goals, values, and preference for MS treatment and could potentially be used to help patients communicate their preferences to their clinician. | **COMMUNICATION/ PREFERENCES:** Asking patients what they value often ineffective because patients may not have pre-formed ideas about what is most important to them and may need help applying deeply held values to clinical decisions.  37% of participants reported not routinely sharing their goals and priorities with their clinician (p.264). Most participants (75%) reported it would be very helpful/extremely helpful if MS clinician were aware of treatment goals and priorities and 19% that it would be somewhat helpful; none felt uncomfortable discussing this (p.264/265).  Many study participants who wanted to discuss their treatment preferences did not routinely do so. Physicians can encourage or obstruct patient involvement (p.265)  **RELATIONSHIP:** Patients are more active when interacting with physicians who engage in partnership building and supportive talk that legitimizes patient’s perspective and creates expectations and opportunities for the patient to discuss their needs and concerns (p. 266). |
| Driedger (2017) [4] | Explores discussions between PwMS and their neurologists about CCSVI against a backdrop of calls for SDM. | Qualitative study (descriptive)  N=69 | PwMS reported a variety of experiences when attempting to discuss CCSVI with their neurologist. Clinicians noted that being respectful of patient emotions, values, and hope was also key to maintaining good relationships. | **RELATIONSHIP:** Long-term relationship with a neurologist may be a source of conflict/dissatisfaction due to poor communication and information sharing. PwMS want more active and autonomous roles, less paternalism.  **COMMUNICATION:** Some patients said that *‘a core problem was that neurologists just generally lack a capacity for good communication and compassionate care’* (p. 10)  *“How things are said are as important as what is being said”* (p. 10). The importance of listening and being open/authentic: *“it has to be real”* (p. 11). |
| Efendi (2018) [5] | What type of decision-making model do MS patients want? | Case studies | In theory, patients want SDM; in practice, most rely more on MD to make decisions. Education may be related to increased SDM. | **INFORMATION:** *“the more the patient gets information about the disease, the more their participation in treatment decisions increase”* (p. 511). |
| Lowden et al (2014) [6] | What is the experience of treatment decision-making in individuals with relapsing-remitting MS? | Qualitative study  (Colaizzi’s (1978) phenomenology)  N=9 | All individuals described a core theme in which decision making about treatment was part of a process of coming to a ‘‘redefined self.’’ This core theme included reflections about self-image, quality of life, goals, and being a person with MS. | **LOSS/ACCEPTANCE:** Participants described decision-making as part of a process of coming to a *‘redefined self’* (p. 17). The process of redefinition started diagnosis of MS and becoming a person with MS (p 17). Thoughts about treatment hinged on reflections about self-image… their life and career goals, and their present and future QOL’ (p. 17). Part of the redefinition process included ‘acknowledging the illness as part of oneself’ (p. 18). One participant described this gradual process: *“it’s [MS] in the house, it’s part of me, but it’s still outside. I’ll have to invite it in . . . “*(p. 18). One participant stated, *‘MS is still not a big part of my life’* (p. 19) therefore put off making decisions about treatment.  ‘Participants struggled with fear, uncertainty and ambiguity of the illness’ and its treatment (p.18). Some participants were proactive about treatment, whereas for others, *“real or anticipated symptoms were not enough to tip the balance in favour of starting therapy* “(p.19)  *“Those who had ‘invited the illness in’ were, in many cases, ready to reflect on treatment to maintain a life they valued. Those who had kept the illness ‘out’ demonstrated a reluctance to consider treatment. It was when they perceived that the illness threatened their quality of life, goals, and self-image that they reconsidered their treatment options’* (p. 20). *‘a reappraisal seemed to occur’* (p. 20).  Decision-making for MS is *‘inextricably woven in the experience of the illness itself’* (p. 20).  The core theme of *‘redefining self’ presents an important aspect to consider; … individuals would not consider treatment until this redefinition of self was done’* (p. 21).  A process of redefining self supports other researchers’ findings that the sense of self-changes after being diagnosed with MS (see p. 21).  *‘symptoms alone did not influence the decision to accept treatment until those symptoms affected perceived QOL or threatened their sense of self’* (p. 21).  *“Choosing a treatment [or choosing to engage in ACP] meant acknowledging the diagnosis, picturing themselves as someone with MS, and imagining being on treatment’* (p. 21).  **FAMILY:** *“Family members provided support, encouragement, and often, filtered information about MS and its treatments when the participant was trying to cope with the treatment decision”* (p.19)  *“Some participants described working as team with family … but ultimately made their own decision about how to proceed”* (p. 19).  **CONTEXT:** Some participants identified MS as a stigma (p. 19). Previous experiences with illness and treatment influenced participants’ decision-making (p. 19)  **TIMING:** *‘Participants felt ill-equipped to make treatment decisions early in the disease trajectory’ (p. 19), as it was ‘overwhelming’* (p. 19). Patients need time to make sense of the diagnosis. But after initial hesitancy, many participants *‘wanted to take a more active role in their treatment choice after having time to learn about the illness and the treatment options available’* (p. 20).  This study raises important questions about appropriate timing of discussions. For instance, those participants who had not reached a point of considering MS as part of their identity were unwilling to receive information, education, or decision support relating to … therapies (p. 22). Experiencing a *‘new illness-related event’* (p. 22) may be catalyst to encourage discussions about decisions.  **RELATIONSHIP:** The necessity of ‘goodness-of-fit’ between their decision-making styles. Also mentioned not taking hope away (p. 20).  **INFORMATION:** Participants valued comprehensive information from HCP, but also wanted the lived experience and *‘peer evidence’ of others’* (p. 22). |
| Lustig et al. 2018 [7] | Relationship between psychosocial adjustment and dysfunctional career thoughts for adults with MS. | Quantitative study  N=94 | Higher levels of depression were associated with higher levels of decision-making confusion and commitment anxiety. | **LOSS/ACCEPTANCE:** *“Individuals with MS experienced a number of significant adjustment problems related to the diagnosis on-going impact of the illness”* (p. 113). Individuals with MS experience stress related to (a) family disruption and caregiver burden; (b) cognitive limitations; (c) the quality of relationships; (d) illness uncertainty; and (e) pessimism (p 113). But individuals with MS adjust over time (p. 113).  **OTHER:** Depression was linked to decreased ability to make decisions: *“depression can lead to errors in thinking that affect the ability to make effective … decisions”* (p. 118) therefore it may be necessary to treat depression before engaging in decision-making with PwMS. |
| Golla et al. 2014 [8] | To investigate how severely affected MS patients and their HC professionals perceive palliative care; to determine how to better approach these patients about this topic. | Qualitative study  (Content analysis)  N=38 (15 patients and 23 HCP) | MS patients were mostly unfamiliar with the term ‘palliative care’ or were aware of it only in relation to cancer and dying. They did not view it as relevant to themselves.  HCP predominantly associated palliative care with dying cancer patients, if familiar with it at all. Most physicians doubted its relevance for neurological patients and denied MS as a cause of death. Most nurses and SW’s regarded palliative care as an opportunity for MS patients. | **RELEVANCE:** Participants *“could not associate palliative care with MS and their own situation at all”* (p. 3). MS patients might have, over time, adjusted to living with their disabilities and thus might not view their condition as ‘terminal’ and in need of palliative care (p. 8). *“one important reason why palliative care was rejected for MS patients was the overwhelming opinion that palliative care would be meaningful only for terminal diseases or for stages typical of various cancers”* (p. 9). The *“illness trajectory of MS and the terminal care phase are less distinct, and doubts exist about whether patients actually truly die from MS”* (p. 9). [note in introduction authors report *“approximately half of all MS patients die from MS itself or related complications”* (p. 1)]  **FEAR OF DEATH/DYING:** Also mentioned the ‘fears surrounding its [palliative care’s] close association with death’ (p. 9). The way death and dying are ‘handled’ (p. 9) is important in management of MS patients and to integrate palliative care. Not having an ACP is a barrier to receiving palliative care (p. 9), and that *“both patients’ and health professionals’ fearful attitudes towards palliative care”* (p. 9) prevent ACP discussions.  **COMMUNICATION:** *“Severely affected MS patients are interested in talking about EoL issues, but doctors hardly ever address this topic”* (p. 9). |
| Clarke et al. (2018) [9] | To investigate how patients and family members make decisions about their future care as their condition deteriorates, with a focus on eating and drinking. | Qualitative study  (thematic analysis)  N=42 | Two main groups of MS patients’ planning styles: those who planned and those who lived in the present.  The long and uncertain trajectory of MS complicates future decision making because patients are considering an unknown future that may be difficult to contemplate [when] they are adjusting to living and coping with a progressive and life-limiting illness. | **AUTONOMY:** Some participants used ACP *“as a way of extending the zone of personal autonomy and involvement in decision-making”* (p. 6).  **INFORMATION:** Information from HCP was not always an important factor in ACP. Some participants made all their own care decisions in advance in keeping with their personality and based on long-term values (p. 7).  **RELEVANCE:** Some participants did not discuss future care: *“particularly in early stages of illness they preferred to focus on the ‘here and now’ rather than thinking about problems that might lie ahead. Decisions about future care were deferred to a later date, sometimes in the apparent hope that eventualities that they could plan for may never arise”* (p. 7). Some preferred not to focus on their future prognosis, which was *‘an active decision, or unconsciously minimising or attempting to ignore it’* (p. 7/8). |
| Thorne et al (2004)a [10] | How do PwMS describe and explain helpful and unhelpful communications in their health care? | Qualitative study  (Interpretive description)  N=12 | In early stages, communication patterns indicate HCP’s tendencies to protect patients from the full truth about their diagnosis and its implications, which stands in contrast with what PwMS say they want, which is full information. | **COMMUNICATION:** Helpful communication is when HCP legitimises and confirms the patient's experience, assists the patient to find the language to describe his/her experience of the illness, is knowledgeable about and considers MS in the context of the patient’s life.  **RELATIONSHIP:** Although actual communication practices were important, the orientation of the HCP’s to the PwMS was even more fundamental (p. 11) Validation of the patient’s experience was helpful, whereas withholding of information was paternalistic and frustrating (p. 11)  Difficulties: providing information evasively rather than directly and general rather than specific (p. 12). The focus of the relationship with HCP must shift from mentorship (at the beginning) to partnership (p. 17).  Context of fear/mistrust based on the long pre-diagnostic phase when PwMS was discounted/patronised by MD.  **OTHER:** To create a context in which empowerment of ‘enablement’ of the patient can occur, chronic diseases might well require longer consultations with different aims than is structured within many GP practises (p. 17). |
| Kalb (2007) [11] | To discuss the meaning attributed by individuals and families to relapses leading to the MS diagnosis and the recommendations for DMT; the adjustments that are made by patients and their families to residual deficits following acute episodes; and recommendations for clinicians on how they might facilitate the adjustment process. | Discussion/opinion | Feelings commonly experienced by people with MS and their loved ones include grief, anxiety, anger, and guilt. | **LOSS/REDEFINITION:** Grief is a normal response to any change in physical or cognitive abilities that interferes with important activities and life goals. People need to grieve over losses before they can begin to engage in productive adaptation and problem solving (p. s29).  Relapses are *“the first step toward acknowledging a major life change—redefining him-or herself as a person with a chronic illness who is faced with an unpredictable future”* (p. S30).  *“People begin to acknowledge the EoL as they knew it and the beginning of life with a chronic illness. This requires a change in self-image—from that of an able-bodied person to someone with a chronic disease”* (p. S30).  A grieving process is needed; impossible to begin planning and problems solving for a new life with MS without first grieving over the loss of the old one (p. S30).  *“When MS relapses produce lasting deficits that interfere with the ability to function in major life roles, the patient is faced with the need to redefine self: ‘who am I now that I can no longer do what I’ve always done? Or ‘what is my value, what do I still have to contribute to my world?’ or ‘what is my role, what can I do for my family, friends, and colleagues?” (p. S31).*  *One of the key elements in successful transition is being able to grieve over losses—of one’s pre-MS sense of self, of cherished abilities, the way things used to be. The individuals who face the most difficulty with these transitions, who feel the greatest loss of self, control, and independence, are those who decide that they would prefer to stop doing something altogether than to have to do it differently or not as well. Their world gradually grows smaller as they give up more and more of their activities.”* (p. S31)  Denial is an important adaptive mechanism, but may *“interfere with a person’s ability to make informed … decisions … and sets the stage for feelings of failure and guilt when the disease progresses”* (p. S30)  Beginning DMT [or ACP] *“is an acknowledgement of the reality of the diagnosis”* (p. S31).  **AUTONOMY:** *“people living with MS struggle to maintain a feeling of control in the face of an unpredictable disease course and an uncertain future.”* (p. S31). Although planning for the worst while hoping for the best is frightening for many, effective planning and problem-solving early in the disease course help people feel more prepared—and more in control—regardless of what the future holds (p. S32).  **INFORMATION:** *“the first step is to provide patients and family members with information about disease activity and progression”* (p. S32). |
| Foley et al. (2012) [12] | To reflect on a case, narrated through the words of family members, in which a 42-year-old died in hospital from complications of MS. The overall aim is to raise thought-provoking issues to neurologists. | Case study | End of life care in progressive neurological illness is challenging and, despite a growing awareness within the neurology community of initiatives to address these issues, these challenges are likely to become more prominent in the future. An expanding evidence base in areas such as prognosis, symptom control, ethics, service provision, and medical training will inform future progress in this important field. | **RELEVANCE/UNCERTAINTY:** *‘predictability of a neurological disease can be challenging’*. This unpredictability, in turn, can contribute to difficulties in discussion with the patient (p. 246).  **TIMING:** Triggers for ACP may include clinical events that indicate disease progression. Triggers may, however, be difficult to discern, therefore *‘early communication and planning is vitally important in progressive neurological illness’* (p. 246).  **AUTONOMY:** *‘more discussion of these issues, earlier in the disease progression, could have enabled [the patient] and her family to have more control over her management’* (p. 246).  **RELATIONSHIP:** *‘a consistent point of contact with health services—a key worker or key team’* —can help to ensure appropriate involvement of professionals at different stages of a patient’s illness (p. 247).  **COMMUNICATION:** the multidisciplinary team could allow better communication and facilitate/prompt ACP  Discussing death and dying with a young person is difficult, and the potential to take away a person’s hope is a deterrent. |
| Leclerc-Loiselle & Legault (2018) [13] | To describe perceptions of home-based health professionals concerning introduction of a palliative care approach in care trajectory of people living with advanced MS. | Qualitative study  N=13 (RN, SW, OT) | Health professionals reported that they feel a palliative care approach for people living with MS is mandatory; however, they do not feel comfortable integrating it systematically into their care. | **RELATIONSHIP: *“****When you meet someone, and you have a trust bond, you can ask a question, plant a seed, to let the person know you’re open for discussion”* (P. 266).  Social workers also thought that discussing EoL issues too early in the disease trajectory could break the bond of trust between themselves, PwMS and their caregivers (p. 267).  **AUTONOMY:** Professionals felt the introduction of a palliative approach could enable increased control over disease (p. 267). *“Discussing the future allows control. She had choices, things to decide. Physically, she had limitations, but having a voice was reassuring”* (p. 267).  **TIMING:** Unpredictable trajectory of MS as a challenge to the introduction of a pc approach (p. 267). Professionals all considered that the adaptation period of PwMS to their new functional reality following an MS crisis was the most appropriate moment to introduce a palliative approach. For all professionals, the moment right after an MS exacerbation was a turning point, because there has usually been a reflexive process as a result of grieving the loss of previous functional abilities (p. 267).  **FEAR OF DEATH/DYING:** Could lead to the loss of hope from PwMS and could diminish their will power to fight against the disease. Discussing the future was mandatory with PwMS, but HCPs should not dictate discussions with PwMS about death and dying. *“I was scared discussing death. I thought it could trigger a depressive episode, that he would project himself into death, and not into the life ahead of him”* (p. 267).  HCPs also suggested that a PwMS’s needs for information are not always met/discussions do not always occur, due to HCPs fears and difficulties in discussing the future. (p. 269). |
| Rieckmann et al., (2015) [14] | Steering group panel to devise themes requiring action regarding patient engagement in MS. | Discussion/opinion | Engaging MS patients involves a broad, multidisciplinary approach. Patient engagement can be practiced at the macro, meso, and micro levels, and is particularly important in chronic illnesses, which necessitate lifelong therapy.  Improved clinical outcomes shown to equate with patient engagement: improved treatment adherence, faster recovery and reduced mortality rates, as well as reduced healthcare consumption and improved service quality. | Depression may have an inverse correlation with patient engagement  5 categories of factors that influence engagement: patient related (patient’s knowledge and beliefs, demographic characteristics, emotions, and coping style); illness-related (symptoms, treatment plan, patient’s prior experience, illness severity); HCP related (HCP’s knowledge and beliefs, HCP role); healthcare setting-related (primary or secondary care); and task-related (medical knowledge required and whether the required patient behaviour challenges clinicians’ clinical abilities). |
| Eskyte (2019) [15] | To investigate how various dimensions impacting shared decision making are important when PwRRMS make DMT decisions. | Literature review  (critical interpretive synthesis)  N=83  Part of larger study to improve understanding of how PwRRMS weigh up pros and cons of DMTs. | Synthesis of ﬁndings revealed that alongside medical and individual reasoning, contextual circumstances play an important role in making treatment decisions. | The need to view decision-making as a contextually imbued process, rather than a passive exercise done after ingesting information from neurologist.  *“Treatment decisions are flexible and dynamic, re-interpreted as life unfolds alongside patients’ experiences of illness and healthcare”* (p. 376)  **RELATIONSHIP:** Trust is necessary (p. 374); limited trust in medical professionals is a barrier for PwMS (p. 374). Also, time constraints are a barrier for HCP to develop trust with patients  **FAMILY:** *“support provided by family and social networks may indirectly shape treatment decisions”* (p. 375). |
| Solari & Pucci (2019) [16] | To discuss the need for palliative care in MS | Discussion/opinion | An early and simultaneous palliative approach should be a care model for persons with PwMS, and the multidisciplinary MS Care Unit should be fully integrated with palliative care competencies. | **TIMING:** [ACP is] considered most effective when started in a timely fashion, allowing patients, their loved ones and HCPs to proactively address the challenges together, while the patient has the ability to express these views and avoid unplanned care for crisis intervention (p. 1). The use of ‘triggers’, based on the clinical changes and the views of the patient and family, to suggest referral to specialist palliative care have been recommended. |
| Golla et al. (2015) [17] | To identify if MS patients discussed death and dying without being ‘prompted’ (secondary analysis). | Qualitative study  (secondary analysis)  N = 15 (Original study on unmet needs of severely affected MS patients) | Participants did talk about death and dying without being ‘prompted’ (though they were asked about attitudes to palliative care in the original study). | **RELEVANCE:** *“Death is not relevant to me; nobody dies of MS”* (p. 281). Many participants avoided the topic or used euphemisms: *“Death is still far away” or* death is not a (viable) alternative; focused more on *“a duty to keep on living”* (p. 281).  Participants did express wish to have opportunity to address EoL issues’ (p. 283) |
| **Source articles including multiple sclerosis alongside**  **other neurologic or chronic illnesses** | | | | |
| Barnes & Campbell, (2010) [18] | Discussion of palliative care approach in MS and MND for general physicians | Discussion/opinion | People with MS and MND may need palliative care to same extent as people with cancer (p. 21). | **TIMING:** A person with MS or MND may benefit from palliative care when there is an expected lifespan of 6–12 months, if there are distressing symptoms or if there is a need to consider end-of-life planning (p. 21).  Palliative care and neurology teams have expertise in guiding patients to make future care decisions. These conversations should occur before swallowing and communication are lost and be introduced by a member of the health care team who feels comfortable/competent in managing such conversations (p. 23) |
| Campbell (2010) [19] | Discusses importance of shifting goals towards a palliative approach as MS/PD become more advanced. | Discussion/opinion | Importance of communication and listening to the patient and family.  Highlights difficulties with prognostication and life expectancy and speaks to timing of ACP discussions. | **COMMUNICATION**: Importance of listening to patient and family  **UNCERTAINTY**: *“the unpredictable disease trajectory makes planning for the terminal event particularly difficult”* (p. 290).  **TIMING:** *‘Broaching the topic of life-prolonging treatments is best done when the patient is relatively well and still has capacity.’* (p. 291) |
| Hamann et al (2010) [20] | Do patients want to participate in decision-making and are physicians able to predict patient preferences? | Quantitative study  N = 102 in-patients with multiple sclerosis and 101 in-patients with schizophrenia or schizo-affective disorder (SAD) | Most patients do want to participate in decision-making (pts with MS more so than patients with schizophrenia/SAD). | **RELATIONSHIP:** *“participation in treatment decisions was stated to be related with one’s trust in the physician”* (p. 310). |
| Oliver, et al. (2016) [21] | To establish an evidence-based consensus for palliative and EoL care for patients with progressive neurological disease, and their families. | Literature review | Limited evidence overall but increasing evidence that palliative care and a multidisciplinary approach to care do lead to improved symptoms and quality of life of patients and their families. Main areas for recommendations are in early integration of palliative care, involvement of the wider multidisciplinary team, communication with patients and families including ACP, symptom management, EoL care, carer support and training, and education for all professionals involved in the care of those patients and families.  There is a pressing need for increased collaboration between neurology and palliative care. | **COMMUNICATION:** The telling of the diagnosis of a progressive neurological disease sets the agenda for the later care of the patient and family.  A survey amongst severely affected MS patients expressed their wish to discuss progression of their disease with their doctors and doctors who avoided such discussions were less empathetic (p. 6). Families do appreciate honesty and awareness of deterioration (p.6).  **TIMING:** Early ACP strongly recommended, especially when impaired communication and cognitive deterioration are possible as part of disease progression.  Within the care of multiple sclerosis, early involvement of palliative care has been shown to improve symptom management and improve patient and family satisfaction (p. 6). Palliative care should be considered early in the disease trajectory, depending on the underlying diagnosis (p. 6). |
| Walter et al (2019) [22] | Questionnaire focused on consultant neurologist’s daily practice concerning timing and content of discussions on treatment restrictions with patients suffering from HGG, PD or MS. Also inquired about education and training in discussing these issues. | Quantitative study  (online survey)  N=125 (neurology consultants and residents in NL) | ACP discussions occur earlier in patients with HGG than PD or MS. However, in all 3 diseases, these discussions usually occur when significant physical and cognitive decline has become apparent and commonly mark the initiation of EoL care.  Physicians expressed a need for more training about EoL discussions. | **TIMING:** ACP discussions in MS generally do not occur until terminal phase, despite recommendations to begin discussions early due to potential for cognitive decline.  *“Significant cognitive decline was the most important trigger for the respondents to advance discussions, followed by physical decline, and the terminal phase”* (p. 5/6).  PwMS expect HCP to initiate discussions about ACP as an adjunct to usual care (p. 6). Uncertainty about optimal timing often causes postponement of discussions” (p. 7).  **AUTONOMY:** Patients want to be included in process and want information to be able to make well-considered decisions, which gives them a feeling of having choice and control over their treatment (p. 6). |
| Thorne et al. (2004)b [23] | To examine commonalities and variations across disease categories to develop some beginning conclusions about the general ﬁeld of health care communication in chronic illness. | Qualitative study (ID)  N=38 persons with chronic illness; 12 with MS | Across each of these conditions, the patients explained the importance of health care communication at three distinct levels: courtesy, respect, and engagement (p. 301). | **RELEVANCE/UNCERTAINTY:** A central feature of disease world of MS is complexity inherent in its diagnostic process (p. 302).  **RELATIONSHIP:** Once individuals are diagnosed, they are therefore already highly guarded in their relationships with health care providers and hesitant to accept professional advice, particularly where it is pessimistic (p. 303). For this group of patients, engagement in constructive on-going conventional health care relationships is less common but highly valued when it occurs. More typically, persons with MS are attuned to courtesy dimension of health care communication assuming that, in its absence, respect and engagement are unlikely. They report highly adversarial responses in communicating with health care professionals, except when they are dependent upon professional’s goodwill and feel obliged to feign gratitude and cooperation (p. 303). |
| Paterson et al. (2002) [24] | To investigate the everyday self-care decision-making of individuals with chronic illness for the purpose of developing a comparison of decision-making processes between chronic diseases and to identify criteria by which persons with various chronic conditions evaluate the quality of self-care decisions. | Qualitative study  (ID with modified think-aloud and focus groups)  N=21; 7 participants each with DMT2; HIV/AIDS, or MS | Although participants shared similar elements in their self-care decision-making, they differed in the perceived meaning and significance of their decisions, depending on disease-specific attributes relating to timeliness, biomarkers, interaction within a social context, the construction of healthy practices, and available relevant information. | **LOSS:** For PwMS … decisions forced by a change in mobility (to accept a wheelchair) or career (to take leave from work) were emotionally charged in that they implied submitting to the progression of the disease rather than merely accommodating a temporary setback |
| **Source articles focused on specific neurological illnesses**  **other than multiple sclerosis** | | | | |
| Murray et al. (2016) [25] | To investigate caregiver perspectives on the acceptability and impact of advance care planning for patients with motor neuron disease and caregivers. | Qualitative study  (narrative synthesis)  N=18 former caregivers of deceased patients with MND | Four global themes emerged: Readiness for death, Empowerment, Connections and Clarifying decisions and Choices. Many felt the letter of future care was or would be beneficial, engendering autonomy and respect for patients, easing difficult decision-making and enhancing communication within families. However, individuals’ ‘readiness’ to accept encroaching death would influence uptake. Appropriate timing to commence advance care planning may depend on case-based clinical and personal characteristics. | **RELEVANCE:** Readiness for death: **acceptance**: completing ACP was easier for patients and caregivers who accepted encroaching death (p. 473). Some caregivers felt that documenting wishes enabled patients to ‘let go’ [thus facilitating acceptance] (p. 474).  *“It prepares you for what you yourself can see, but refuse to accept – so yes, it was helpful to me and to my son”* (p. 474).  **TIMING:** Several participants thought that if initiated too soon, completing the LFC (letter of future care) might be too confronting, emotional, conflictual and would diminish hope (p. 474).  Triggers for end-of-life discussions, including when it first becomes clear patient has life-limiting advanced progressive illness and critical change points during illness trajectory. However, previous qualitative findings suggest ACP completion and timing less significant when caregiver and patient wishes are concurrent (p. 476).  **EMPOWERMENT/AUTONOMY:** Many caregivers felt that the LFC gave, or would have given, patients’ autonomy, feelings of control and courage to say what they wanted. *“… the letter just gave him control of his life. That’s what his decision was that he still had control and dignity …”* (p. 474).  **FAMILY:** Caregivers also described how the LFC helped them negotiate with the rest of the family; “I wasn’t forcing this issue; this is what he wanted” (p. 475).  Reduce the decisional burden and help caregivers avoid regret (p. 475).  **COMMUNICATION:** Several participants found the LFC assisted by opening family communication or composing the LFC was catalyst for communication between them (p. 475). generated meaningful discussion and facilitated agreement in several families (p. 475). |
| **Source articles focused on unspecified**  **neurological illnesses** | | | | |
| Seeber et al. (2012) [26] | To explore ACP in acute and chronic stroke: “at what moment in the course of the neurologic disease are treatment restrictions; which factors determine the timing; who initiates and participates in the discussion? | Literature review | Strong influence of family on treatment decisions; ACP triggered by crises and left until end. | **FAMILY:** Importance of including the family in the discussions  “involving families in discussion appears to improve acceptance of decisions for both patient and caregiver”  **TIMING:** ACP discussions are triggered by life-threatening situations: ‘generally, discussions are postponed until very last minute, and often family wishes are privileged over patients (especially in US).  **RELATIONSHIP:** They say the neurologist is ‘best poised’ to have discussion with patients, but do not elaborate on why, other than “their detailed knowledge of neurologic diseases and possible complications” (p. 595). |
| Kent (2015) [27] | To provide an overview of ACP and its use when caring for people with progressive neurological conditions. | Discussion/opinion | Advance care planning for people with progressive neurological conditions should be a fundamental, but voluntary, component of care. | **LEGAL STATUS (UK):** While an advance care plan is not legally binding, it does have a legal standing and its contents should be considered and taken into account if an individual is not able to make his or her wishes known in the future and a best interest decision has to be made (p. 53).  **BENEFITS/AUTONOMY:** The development of an ACP supports patient choice and can alleviate concerns. It has been linked to feelings of increased individual autonomy and control, improved quality of life and coping and reduced fear of death and the mechanism of death.  **COMMUNICATION:** Barriers to ACP include reluctance on the part of HCP’s to initiate discussions in case they cause distress. An ACP can also contribute to effective communication within a multidisciplinary team, with all involved being aware of and committed to fulﬁlling the individual’s choices (p. 56).  **TIMING:** Patients with a range of conditions have often welcomed early discussions, indicating that they felt the conversations should be initiated by healthcare professionals and that they wanted this involvement and support (p. 56).  **UNCERTAINTY:** The disease trajectory and rate of progression for people with a progressive neurological condition will vary. However, there may be interventions to discuss that are common to all patients (p. 56).  **OTHER: CULTURE:** Cultural values and beliefs may deter people from talking about death and dying (p. 56) |
| Lennard (2018) [28] | To explore the role of nurses in assisting people with HD in ACP. | Case study | Recommends ‘in the moment’ decisions focused on ‘now’ as opposed to the individual’s former self; the “chief object of ACP should be to prepare people and their surrogates to work with health professionals to make the best in-the-moment decisions” (p. 1265). Advance decisions cannot be genuinely informed decisions, because the competent person must anticipate a state they have never experienced: that of incapacity” (p. 1263). But some assert that an individual who has lost capacity may have different interests from their former selves when they had capacity”. | **TIMING:** Important to *“begin discussion at the earliest opportunity, when the individual is still able to participate”* (p. 1261).  People are reluctant to talk about EoL care, because death is *‘frequently regarded as a cultural taboo’* (p. 1261)  **FAMILY:** Having supportive families was an important factor in decision-making about future care; with individuals feeling that the burden was shared. Family involvement provided the individual agrees, enables the views of their loved ones to be considered’ (p. 1261).  **AUTONOMY:** England is a country with *‘strong libertarian traditions and respect for individual wishes’* (p. 1263); as such, ACP may be viewed as a way of *“enhancing patient autonomy”* (p. 1263). |
| Gofton et al. (2018) [29] | To develop a conceptual understanding of the specific characteristics of palliative care in neurology and the challenges of providing palliative care in the setting of neurological illness. | Qualitative study  (GT with thematic analysis)  N=3 dyads (interviews)  4 focus groups (HCP’s) Actual participant numbers not provided | Specific characteristics of neurological disease that affect palliative care: i) timelines of disease progression; ii) barriers to communication arising from neurologic disease; iii) variability across disease progression; iv) threat to personhood arising from functional and cognitive impairments related to neurologic disease.  Three key challenges: i) uncertainty with respect to prognosis, support and trajectory; ii) inconsistency in information, attitudes, and skills among caregivers, including families; iii) existential distress specific to neurological disease, including emotional, psychological, and spiritual distress resulting from loss of function, autonomy and death | **TIMING:** *“since the course of many neurological diseases lead to significant dysfunction, many patients require timely and effective planning regarding goals of care, substitute decision-makers, ACP and EoL decisions …”* (p. 225). Uncertain trajectory is a barrier to palliative care  **COMMUNICATION:** Difficulties from neurologic disease (aphasia) mean that designating an SDM is ‘the most important part of ACP” (p. 227), which *“implies an educational requirement early in the disease trajectory”* (p. 227). “neurology residents indeed feel unprepared to manage death and dying” (p. 230).  **LOSS (OF SELF)** *“neurological disease often involves earlier impairment or loss of cognitive function as well as substantial, long-term limitation of function and autonomy: ‘[neurology] patients face threats to their very personhood that are perhaps a little bit different than some other kinds of illnesses”* (p. 227). Patients often experience *“symptoms in the whole psychosocial realm and the loss of personhood and trying to give this patient their dignity back”* (p. 227)  *“Sense of self and purpose are especially undermined through limitations of autonomy and self-expression common in neurologic pathologies … which also impacts caregivers”* (p. 227)  **EXISTENTIAL DISTRESS** *“For patients with life-limiting neurologic disease, the primary source of existential distress may not be death, but the progressive and irreversible loss of function and autonomy”* (p. 229).  Also important is the “existential distress that arises from progressive loss of function, autonomy, and personhood” (p. 229).  **UNCERTAINTY/RELEVANCE:** is *“a substantial barrier to the effective delivery of neuro-palliative care”* (p. 227). *“for the patient and family, [it] can be quite hard when you’re discussing EoL with the patient because they have always felt that their disease isn’t … an end-stage disease or a terminal disease”* (p. 227). Uncertainty also placed strain on families, who expressed difficulty understanding the expected trajectory of illness and role for palliative care (p. 228).  **INFORMATION/TRUST:** Inconsistency of information *“could also erode patient and family trust in the health care team”* (p. 228). |
| Vishnevetsky et al. (2019) [30] | To explore attitudes toward and knowledge about palliative and end-of-life care among patients, families, nurses, and doctors in a specialized neurological institute. | Mixed methods: 78 surveys and 21 qualitative interviews (thematic analysis)  Quantitative N=21 patients; 16 family members; 28 doctors and 13 nurses  Qualitative N=21 (3 patients; 10 family members; 2 nurses; 6 MDs). | A substantial need exists for palliative care in the neurological institute. Key themes: transparency of communication about prognosis and end-of-life choices in neurological disease.  Barriers to transparency in patient-physician communication included (1) expectation of cure; (2) physician’s lack of training in communication and end-of-life care; (3) a paternalistic culture; and (4) the nature of neurological diseases  Providers felt uncomfortable with basic palliative care skills such as initiating difﬁcult conversations or breaking bad news. Because these skills are fundamental to developing a trusting clinician-patient relationship, the inclusion of basic palliative care and communication training into the Peruvian health system needs to be a priority (p. 255). | **COMMUNICATION:** *“we found an ambivalence concerning the transparency of communication: on the one hand, participants neither had much experience with advance directives or the concept of treatment choice, nor felt comfortable with end-of-life conversations”*. Several barriers to transparency were identiﬁed; these included a focus on cure, paternalism, lack of physician training, and conversation barriers speciﬁc to neurological disease (p. 252). Inexperience with *‘‘the conversation’’* and advance directives. Few family members reported ever having a conversation regarding their health care preferences for end of life (p. 253). Discomfort with *‘‘the conversation’’* (p. 253).  Participants suggested that openly discussing difﬁcult topics might be helpful. Several participants became emotional during the interview, but then ended by saying they found the discussion during the interview itself beneﬁcial. (p. 253). Despite not having discussed these issues with their respective families, some participants revealed that they had thought about the concepts of death and dying for themselves and showed some openness to the discussion (p. 253).  Physicians feared that patients might feel abandoned if they gave them no hope for a cure (p. 254).  **TIMING:** Some family members also stated that they felt it was important to discuss end-of-life issues ahead of time, but their family was too uncomfortable to engage with them (p. 253).  **LEGAL STATUS:** One doctor described a lack of legal clarity for the use of advance directives or do-not-resuscitate orders and their documentation (p. 253)**.** |
| Boersma et al. (2014) [31] | To discuss the role of palliative care in neurology. | Discussion/opinion | To provide a practical starting point in palliative medicine for neurologists by answering the following questions: (1) What is palliative care and what is hospice care? (2) What are the palliative cares needs of neurology patients? (3) Do neurology patients have unique palliative care needs? (4) How can palliative care be integrated into neurology practice? | **TIMING:** Neurology patients have a different disease trajectory than cancer patients. Contrary to the common clinical perception that patients do not want to discuss advance directives, patients often cite their expectation for physicians to initiate this discussion when asked about barriers to planning for the future, and patients who engage in end-of-life conversations with their doctors report greater satisfaction with their care. e1,e2 Notably, having conversations about death and dying with patients lowers the risk of aggressive treatment at the end of life (p. 564).  **FAMILY:** The physical and cognitive disabilities associated with neurologic illness also contribute to feelings of being “useless” or a “burden” and may contribute to higher rates of demoralization. |
| Taylor et al. (2019) [32] | To compare characteristics and needs of inpatients with neurologic disease to those with cancer referred for palliative care consultation. | Quantitative study  N = 70,655 palliative care consultations  (dataset) | Patients with neurologic disease as a reason for PC consultation are more in need of EoL care planning and are more likely to die in hospital than those with cancer. The most common reason for PC consultation in all patients was assistance with goals of care and ACP. | **COMMUNICATION:** Eﬀective communication about goals of care is fundamental in the care of all patients with a serious illness. Discussing the patient’s goals and values is a speciﬁc skill that all clinicians need to learn, and a hospitalization represents an important opportunity and need to engage in such a conversation. (14). This need may be especially true for hospitalized patients with neurologic disease, who, compared to patients with cancer, were more commonly referred to PC for transitions to comfort measures only and withdrawal of life-sustaining treatment and had a higher in-hospital mortality.  Many [neurologists] still feel uncomfortable having potentially emotionally charged conversations with patients or families about their treatment options. Because neurologists care for many patients with serious illness in the inpatient and outpatient settings, we need to prioritize eﬀorts to teach communication skills to neurology trainees and research to help us adjust what we know to be (e1979).  **TIMING:** However, for patients with progressive neurologic illness such as ALS, PD, or MS, late engagement with PC teams in the ICU represents a missed opportunity to clarify goals of care and potentially avoid unwanted intensive care and death in the hospital. |
| **Source articles focused on disability** | | | | |
| Mitchell (2017) [33] | To characterise perspectives of individuals with serious physical disabilities receiving care from two different healthcare delivery settings on the value of AD and ACP. | Qualitative study  (thematic analysis)  N=25 (adults with serious ambulatory disabilities) | Five organizing themes emerged as follows: 1) AD is a right versus responsibility, 2) past medical experiences inﬂuence ACP engagement, 3) ACP requires relationship-centred decision support, 4) concerns for care after death, and 5) suggestions for improving ACP experiences. | **FAMILY:** Some participants believed AD constituted a personal responsibility to their family members and friends and were concerned about the ﬁnancial or emotional burden their loved ones could confront if charged with making decisions around life-sustaining care for the individual (p. 128).  **AUTONOMY:** By contrast, other participants felt AD constituted a responsibility to them, a healthcare right necessary to protect. AD represented asserting agency over his care.  **PREVIOUS EXPERIENCE:** Participants wished to engage in a relationship-centred approach to ACP, yet voiced hesitation due to experiences of signiﬁcant medical bias and mistreatment, typically surrounding judgments of their quality of life. Cautious approaches to ACP, concerned with confronting further stigma and discrimination due to past medical experiences. Many participants felt medical practitioners considered the lives of PwD less valuable and therefore did not trust them to make care decisions aligning with their best interests: *‘I don’t trust the medical system to do what’s best for me. That’s my experience overall so. When I’m in a medical setting [I] make sure … decisions are in my best interest … because when you go into the hospital, that means you could die. But it’s the idea that the medical system can have control over me’* (p. 129).  **FEAR:** These participants spoke from a deep sense of vulnerability felt by many PwD in healthcare settings, resulting in apprehension to engage in end-of-life conversations for fear that care may be withheld due to perceived lesser quality of life.  We just need a paradigm shift in society so that death becomes part of our life experience as opposed to just sending people to a hospital to die and just being shocked when they do instead of getting the proper palliative hospice care (p. 131).  Most of our participants expressed some level of fear of differential treatment by medical providers between disabled and nondisabled individuals, and some feared this would translate into care being withheld at the end of life. [power]  **RELATIONSHIP:** a trusting relationship with providers as a prerequisite to ACP discussions (p. 130). Opinions varied on who was the ‘best’ person with whom to discuss ACP: HCP  (MD or RN) or peers, family, friends. The main criteria, though, was that a trusting relationship was foundational.  **TIMING/TIME:** participants thought conversations concerning ACP and AD should be extended, both in time and in frequency and revisited yearly. (p. 131).  **COMMUNICATION:** One participant believed her physician was uncomfortable discussing this topic, alluding to insufﬁcient training and familiarity with ACP among healthcare professionals (p. 132). |

**Key to abbreviations**

**ACP:** Advance care planning, **AD:** Advance directive, **ALS:** Amyotrophic lateral sclerosis, **DMT:** Disease modifying therapy, **EoL:** End of life, GP: General practitioner/family doctor, **HCP:** Health care professional, **ICU:** Intensive care unit, **MND:** Motor neurone disease, **PwD**: People with disability; **PwMS:** Person with MS, **PwRRMS:** Person with relapsing–remitting MS.

1. Buecken R, Galushko M, Golla H, Strupp J, Hahn M, Ernstmann N, et al. Patients feeling severely affected by multiple sclerosis: How do patients want to communicate about end-of-life issues? Patient Education and Counseling. 2012;88(2):318-24. doi: <https://doi.org/10.1016/j.pec.2012.03.010>.

2. Chen H, Habermann B. Ready or not: planning for health declines in couples with advanced multiple sclerosis. The Journal of neuroscience nursing : journal of the American Association of Neuroscience Nurses. 2013;45(1):38-43. doi: 10.1097/JNN.0b013e318275b1f9. PubMed PMID: 23291870.

3. Col NF, Solomon AJ, Springmann V, Ionete C, Alvarez E, Tierman B, et al. Evaluation of a Novel Preference Assessment Tool for Patients with Multiple Sclerosis. Int J MS Care. 2018;20(6):260-7. Epub 2018/12/21. doi: 10.7224/1537-2073.2017-021. PubMed PMID: 30568563; PubMed Central PMCID: PMCPMC6295881.

4. Driedger SM, Maier R, Marrie RA, Brouwers M. Caught in a no-win situation: discussions about CCSVI between persons with multiple sclerosis and their neurologists - a qualitative study. BMC Neurol. 2017;17(1):176. Epub 2017/09/09. doi: 10.1186/s12883-017-0954-7. PubMed PMID: 28882115; PubMed Central PMCID: PMCPMC5590111.

5. Efendi H, Boz C, Karabudak R. Evaluating Treatment Decision for Multiple Sclerosis: Real Life and Patient Experiences. Noro Psikiyatr Ars. 2018;55(Suppl 1):S10-S4. doi: 10.29399/npa.23164. PubMed PMID: 30692848.

6. Lowden D, Lee V, Ritchie JA. Redefining self: patients' decision making about treatment for multiple sclerosis. J Neurosci Nurs. 2014;46(4):E14-24. Epub 2014/05/31. doi: 10.1097/jnn.0000000000000064. PubMed PMID: 24875289.

7. Lustig DC, Xu YJ, Strauser DR, MacKay MM. The Relationship Between Career Thoughts and Adjustment for Individuals With Multiple Sclerosis. Rehabilitation Counseling Bulletin. 2017;61(2):112-20. doi: 10.1177/0034355217709457.

8. Golla H, Galushko M, Pfaff H, Voltz R. Multiple sclerosis and palliative care - perceptions of severely affected multiple sclerosis patients and their health professionals: a qualitative study. BMC Palliat Care. 2014;13(1):11. Epub 2014/03/20. doi: 10.1186/1472-684x-13-11. PubMed PMID: 24641905; PubMed Central PMCID: PMCPMC3995147.

9. Clarke G, Fistein E, Holland A, Tobin J, Barclay S, Barclay S. Planning for an uncertain future in progressive neurological disease: a qualitative study of patient and family decision-making with a focus on eating and drinking. BMC Neurol. 2018;18(1):115. Epub 2018/08/18. doi: 10.1186/s12883-018-1112-6. PubMed PMID: 30115018; PubMed Central PMCID: PMCPMC6094897.

10. Thorne SE, Harris SR, Mahoney K, Con A, McGuinness L. The context of health care communication in chronic illness. Patient Educ Couns. 2004;54(3):299-306. Epub 2004/08/25. doi: 10.1016/j.pec.2003.11.009. PubMed PMID: 15324981.

11. Kalb R. The emotional and psychological impact of multiple sclerosis relapses. J Neurol Sci. 2007;256 Suppl 1:S29-33. Epub 2007/03/14. doi: 10.1016/j.jns.2007.01.061. PubMed PMID: 17350045.

12. Foley P, Hampton J, Hampton A, Hampton R, Oleksy D, Oliver D, et al. Lesley's story: a case report, and discussion of challenges faced in end-of-life care for progressive neurological disease. Pract Neurol. 2012;12(4):244-8. Epub 2012/08/08. doi: 10.1136/practneurol-2012-000263. PubMed PMID: 22869768.

13. Leclerc-Loiselle J, Legault A. Introduction of a palliative approach in the care trajectory among people living with advanced MS: perceptions of home-based health professionals. Int J Palliat Nurs. 2018;24(6):264-70. Epub 2018/06/23. doi: 10.12968/ijpn.2018.24.6.264. PubMed PMID: 29932829.

14. Rieckmann P, Boyko A, Centonze D, Elovaara I, Giovannoni G, Havrdova E, et al. Achieving patient engagement in multiple sclerosis: A perspective from the multiple sclerosis in the 21st Century Steering Group. Mult Scler Relat Disord. 2015;4(3):202-18. Epub 2015/05/27. doi: 10.1016/j.msard.2015.02.005. PubMed PMID: 26008937.

15. Eskyte I, Manzano A, Pepper G, Pavitt S, Ford H, Bekker H, et al. Understanding treatment decisions from the perspective of people with relapsing remitting multiple Sclerosis: A critical interpretive synthesis. Mult Scler Relat Disord. 2019;27:370-7. Epub 2018/11/27. doi: 10.1016/j.msard.2018.11.016. PubMed PMID: 30476873.

16. Solari A, Pucci E. There is an urgent need for palliative care specialists in MS - Yes. Mult Scler. 2019;25(13):1710-1. Epub 2019/07/26. doi: 10.1177/1352458519841827. PubMed PMID: 31343953.

17. Golla H, Galushko M, Strupp J, Karbach U, Pfaff H, Ostgathe C, et al. Patients Feeling Severely Affected by Multiple Sclerosis: Addressing Death and Dying. OMEGA - Journal of Death and Dying. 2015;74(2):275-91. doi: 10.1177/0030222815598443.

18. Barnes J, Campbell C. Palliative care in multiple sclerosis and motor neurone disease. Br J Hosp Med (Lond). 2010;71(1):21-5. Epub 2010/01/19. doi: 10.12968/hmed.2010.71.1.45968. PubMed PMID: 20081637.

19. Campbell CW, Jones EJ, Merrills J. Palliative and end-of-life care in advanced Parkinson's disease and multiple sclerosis. Clin Med (Lond). 2010;10(3):290-2. Epub 2010/08/24. doi: 10.7861/clinmedicine.10-3-290. PubMed PMID: 20726466; PubMed Central PMCID: PMCPMC5873561.

20. Hamann J, Mendel R, Schebitz M, Reiter S, Bühner M, Cohen R, et al. Can Psychiatrists and Neurologists Predict Their Patients' Participation Preferences? The Journal of Nervous and Mental Disease. 2010;198(4):309-11. doi: 10.1097/NMD.0b013e3181d6128c. PubMed PMID: 00005053-201004000-00011.

21. Oliver DJ, Borasio GD, Caraceni A, de Visser M, Grisold W, Lorenzl S, et al. A consensus review on the development of palliative care for patients with chronic and progressive neurological disease. Eur J Neurol. 2016;23(1):30-8. Epub 2015/10/02. doi: 10.1111/ene.12889. PubMed PMID: 26423203.

22. Walter HAW, Seeber AA, Willems DL, de Visser M. The Role of Palliative Care in Chronic Progressive Neurological Diseases—A Survey Amongst Neurologists in the Netherlands. Frontiers in Neurology. 2019;9(1157). doi: 10.3389/fneur.2018.01157.

23. Thorne S, Con A, McGuinness L, McPherson G, Harris SR. Health care communication issues in multiple sclerosis: an interpretive description. Qual Health Res. 2004;14(1):5-22. Epub 2004/01/17. doi: 10.1177/1049732303259618. PubMed PMID: 14725173.

24. Paterson B, Thorne S, Russell C. Disease-specific influences on meaning and significance in self-care decision-making in chronic illness. Can J Nurs Res. 2002;34(3):61-74. Epub 2002/11/12. PubMed PMID: 12425011.

25. Murray L, Butow PN, White K, Kiernan MC, D'Abrew N, Herz H. Advance care planning in motor neuron disease: A qualitative study of caregiver perspectives. Palliat Med. 2016;30(5):471-8. Epub 2016/02/06. doi: 10.1177/0269216315613902. PubMed PMID: 26847526.

26. Seeber AA, Hijdra A, Vermeulen M, Willems DL. Discussions about treatment restrictions in chronic neurologic diseases: a structured review. Neurology. 2012;78(8):590-7. Epub 2012/02/22. doi: 10.1212/WNL.0b013e318247cc56. PubMed PMID: 22351797.

27. Kent A. Advance care planning in progressive neurological conditions. Nurs Stand. 2015;29(21):51-9. Epub 2015/01/22. doi: 10.7748/ns.29.21.51.e9192. PubMed PMID: 25605116.

28. Lennard C. Best interest versus advance decisions to refuse treatment in advance care planning for neurodegenerative illness. Br J Nurs. 2018;27(21):1261-7. Epub 2018/11/21. doi: 10.12968/bjon.2018.27.21.1261. PubMed PMID: 30457382.

29. Gofton TE, Chum M, Schulz V, Gofton BT, Sarpal A, Watling C. Challenges facing palliative neurology practice: A qualitative analysis. J Neurol Sci. 2018;385:225-31. Epub 2017/12/27. doi: 10.1016/j.jns.2017.12.008. PubMed PMID: 29277430.

30. Vishnevetsky A, Zapata del Mar C, Cam JL, Cornejo-Olivas M, Creutzfeldt CJ. Palliative Care: Perceptions, Experiences, and Attitudesin a Peruvian Neurologic Hospital. Journal of Palliative Medicine. 2019;22(3):250-7. doi: doi.org/10.1089/jpm.2018.0196.

31. Boersma I, Miyasaki J, Kutner J, Kluger B. Palliative care and neurology: time for a paradigm shift. Neurology. 2014;83(6):561-7. Epub 2014/07/06. doi: 10.1212/wnl.0000000000000674. PubMed PMID: 24991027; PubMed Central PMCID: PMCPMC4142002.

32. Taylor BL, O'Riordan DL, Pantilat SZ, Creutzfeldt CJ. Inpatients with neurologic disease referred for palliative care consultation. Neurology. 2019;92(17):e1975-e81. Epub 2019/03/29. doi: 10.1212/wnl.0000000000007364. PubMed PMID: 30918095; PubMed Central PMCID: PMCPMC6511082.

33. Mitchell SE, Weigel GM, Stewart SK, Mako M, Loughnane JF. Experiences and Perspectives on Advance Care Planning among Individuals Living with Serious Physical Disabilities. J Palliat Med. 2017;20(2):127-33. Epub 2016/11/05. doi: 10.1089/jpm.2016.0168. PubMed PMID: 27809645.
